# Hypophosphataemia among Severely-malnourished Children: Case Series

**DOI:** 10.3329/jhpn.v30i4.13419

**Published:** 2012-12

**Authors:** Shoji Yoshimatsu, Mohammod Jobayer Chisti, Md. Iqbal Hossain, Md. Munirul Islam, Takashi Fukushima, Yukiko Wagatsuma, Jonathan Harvey Smith, Ryo Sumazaki, Tahmeed Ahmed

**Affiliations:** ^1^Department of Clinical Trial and Clinical Epidemiology, Faculty of Medicine, University of Tsukuba, Tsukuba, Japan; ^2^Department of Child Health, Faculty of Medicine, University of Tsukuba, Ibaraki, Japan; ^3^icddr,b, GPO Box 128, Dhaka 1000, Bangladesh; ^4^Portex Unit of Paediatric Anaesthesia, Institute of Child Health, UCL, UK

**Keywords:** Electrolyte imbalance, Hypokalaemia, Hypophosphataemia, Malnutrition, Sepsis, Bangladesh

## Abstract

Phosphorus is an essential substance in our body, and hypophosphataemia (HP) is well-described in rickets, refeeding syndrome, diabetic ketoacidosis (DKA), and in chronic alcohol-abuse. However, to our knowledge, HP among severely-malnourished children has not been studied in detail, and information on prevalence, severity, and treatment is scarce. Currently, there are only a few published case reports of HP. This case series describes three cases of HP that presented to Dhaka Hospital of the International Centre for Diarrhoeal Disease Research, Bangladesh (icddr,b). Our first case required mechanical ventilation for respiratory distress associated with severe hypokalaemia (K 1.1 mmol/L) and moderate hypophosphataemia (P 2.1 mg/dL). The second case presented with severe sepsis which was associated with symptomatic hypocalcaemia (Ca 1.68 mmol/L), hypokalaemia (K 1.82 mmol/L), and severe hypophosphataemia (P 0.9 mg/dL). The third case presented with pneumonia and sepsis which were complicated by hypokalaemia (K 2.05 mmol/L) and severe hypophosphataemia (P 1.1 mg/dL). Marked lethargy and severe hypotonia were associated with HP in all of these cases. Manifestations of HP are diverse and can occur in association with other electrolyte imbalances, especially among malnourished children. Malnutrition, combined with sepsis, is one of the major killers of children younger than 5 years of age, and both malnutrition and sepsis can cause HP. It is concluded that the underlying causes of morbidity, including HP, should be actively sought and treated to reduce the mortality of children aged below five years.

## INTRODUCTION

During treatment for severely-malnourished children, the principles of management are to reduce mortality by treating hypoglycaemia, hypothermia, infection, and water-electrolyte imbalance (1). Traditionally, electrolyte imbalances, such as hyper or hyponatraemia, hyper or hypokalaemia, and hypocalcaemia, are rapidly diagnosed and treated while hypophosphataemia (HP) has been given less prominence. The important interaction between HP and severe malnutrition among children in developing countries has received little attention, and there are only a few reports from Africa on HP (2-3).

Phosphate is a major anion which is essential for maintenance of human body. It is one of the most important minerals in the formation of human bone, a component of adenosine tri-phosphate (ATP), a necessary substance for nucleic acid synthesis and an essential component of cell membranes and major intra-cellular structures (4). Various manifestations of HP have been described, such as arrhythmias, respiratory failure, seizures, delirium, haemolysis, leukocyte dysfunction, and rhabdomyolysis. Phosphate deficiency can occur because of decreased intake, refeeding, transcellular shifts during treatments, such as for glucose infusion, excessive renal losses, and from multifactorial causes, such as sepsis, dialysis, and alcoholism (5-8).

In this case series, a neglected field of malnutrition, namely hypophosphataemia, is reviewed and discussed in relation to our clinical cases.

## CASE HISTORY

### Case 1

A 24-month old female infant, with weight 6.50 kg, height 72.2 cm (weight-for-height z-score −3.26, weight-for-age z-score −4.62, length-for-age z-score −4.41) was admitted to icddr,b's, Dhaka Hospital. She had a history of watery diarrhoea and vomiting for seven days, fever for six days, severe lethargy for one day, and respiratory distress for two hours. On admission, physical examination revealed a body temperature of 37.0 ºC, heart rate 148/min, respiration rate 48/min, and SpO_2_ 71% (breathing room air). The patient was very lethargic, the eyes were sunken, the oral mucosa was dry, and skin turgor was reduced. Breathing sounds were normal, and there was no heart-murmur. Abdomen was soft and non-tender. Soon after admission, the patient developed profound hypotonia which was associated with an absence of respiratory effort, requiring endotracheal intubation and mechanical ventilation. Laboratory tests revealed mild hyponatraemia (Na 125 mmol/L), mild hypocalcaemia (Ca 1.69 mmol/L), and severe hypokalaemia (K 1.1 mmol/L). A diagnosis of severe electrolyte imbalance with dehydration, severe acute malnutrition, and sepsis was made. Therapy was started with intravenous antibiotics, fluid therapy to correct hyponatraemia, hypocalcaemia, and hypokalaemia, and mechanical ventilation. On the 3^rd^ day of admission, sodium levels recovered to 137.3 mmol/L but potassium and calcium remained low (K 1.73 mmol/L, Ca 1.69 mmol/L), and serum phosphate measurement revealed moderate hypophosphataemia (P 2.1 mg/dL). On the 4^th^ day, the plasma potassium had risen to 2.79 mmol/L, spontaneous respiration had commenced, and weaning from the mechanical ventilator and extubation was possible. A brief spell of post-extubation stridor due to laryngeal oedema was treated with intravenous dexamethasone and nebulized adrenaline. The plasma electrolytes normalized over the following 7 days, and the patient was discharged on the 20^th^ day with the following blood test results: Na 137.2 mmol/L, K 4.41 mmol/L, Ca 2.71 mmol/L, P 3.8 mg/dL.

### Case 2

A 12-month old male infant, with weight 4.8 kg, height 68.9 cm (weight-for-height z-score −6.21, weight-for-age z-score −5.61, length-for-age z-score −2.88) was admitted to icddr,b's, Dhaka Hospital. He had a history of watery stool for five days and high-grade intermittent fever with cough for four days. On admission, physical examination revealed a temperature of 38.7 ºC, heart rate 120/min with normal rhythm, and respiration rate 48/min. In addition, the patient was lethargic, hypotonic, and severely dehydrated. On auscultation of the chest, coarse crackles were found in both lungs, and the heart sounds were normal. The abdomen was soft and non-tender. On admission, biochemical data showed Na 139.1 mmol/L, K 1.82 mmol/L, and P 2.4 mg/dL. The chest x-ray showed bilateral hilar consolidation. Based on the physical examination and laboratory data, the initial diagnosis was pneumonia complicated by sepsis, severe malnutrition, hypokalaemia, and hypophosphataemia. Initial treatment was given with intravenous antibiotics and fluid therapy. In addition, zinc, folic acid, multivitamin, potassium and magnesium supplements were given according to icddr,b's standardized management protocol for severely-malnourished children. On the second day, tetanic spasm was developed as secondary complication due to hypocalcaemia (Ca 1.68 mmol/L). Repeated biochemical results showed continued hypophosphataemia (P 0.9 mg/dL) while sodium and potassium levels were almost normal (Na 133.8 mmol/L, K 3.80 mmol/L). The tetanic spasm was treated with intravenous calcium gluconate and vitamin D. By the 6^th^ day, the patient recovered from fever and was discharged on the 9^th^ day with normal plasma electrolyte levels (Na 136.8 mmol/L, K 4.30 mmol/L, Ca 2.35 mmol/L, P 5.2 mg/dL).

### Case 3

A 9-month old female infant, with weight 5.6 kg, length 65.9 cm (weight-for-length z-score −3.04, weight-for-age z-score −3.29, length-for-age z-score −1.89) presented in icddr,b's Dhaka Hospital. She had a history of soft stool and cough for five days, fever for three days, vomiting for one day, and respiratory distress for 10 hours. On admission, physical examination revealed a temperature of 36.6 ºC, heart rate 118/min, respiration rate 60/min, and blood pressure 60/40 mmHg. In addition, the patient was very lethargic, there was lower chest wall-indrawing and some dehydration. On auscultation, coarse crackles were found in the right lung, and the heart sounds were normal. The abdomen was soft, non-tender, and bowel sounds were present. Chest x-ray showed patchy shadowing in the right lung field, and plasma electrolyte tests revealed hypokalaemia (K 2.05 mmol/L) and hypophosphataemia (P 2.5 mg/dL). Based on the physical findings and laboratory results, an initial diagnosis of severe pneumonia complicated by severe malnutrition, sepsis, hypokalaemia, and hypophosphataemia was made. Initial treatment was given with intravenous antibiotics and fluid therapy, with additional management given according to the icddr,b's standardized management protocol for severely-malnourished children. On the second day, plasma electrolyte analysis revealed a rise in potassium (K 2.87 mmol/L) but a drop in phosphate (P 1.1 mg/dL) levels. By the 7^th^ day of admission, the patient became afebrile, clinically much improved, and the plasma electrolytes were almost corrected (Na 138.3 mmol/L, K 2.93 mmol/L, Ca 2.0 mmol/L, and P 2.8 mg/dL). Unfortunately, the family members removed the patient from the hospital against medical advice at this point.

## DISCUSSION

### Phosphorus concentration in childhood

Phosphorus requirement in childhood is greater than in adults due to the demands of physical growth. Therefore, plasma phosphorus concentration (Table 1) varies with age (4). Defining severe hypophosphataemia is difficult but, typically, levels less than 1 or 1.5 mg/dL can be classified as severe, since clinical manifestations may be encountered at this level. The second and the third cases are classified as severe HP according to this criterion.

**Table 1.** Normal serum phosphorus range during childhood

Age	Normal phosphorus range
0–5 days	4.8–8.2 mg/dL
1–3 years	3.8–6.5 mg/dL
4–11 years	3.7–5.6 mg/dL
12–15 years	2.9–5.4 mg/dL
16–19 years	2.7–4.7 mg/dL

### Pathophysiology and clinical manifestations of hypophosphataemia

Phosphorus is an important component of body tissues and is vital for the normal functioning of blood cells, muscles, and nerves. Hypophosphataemia (HP) results in decreased intracellular ATP and 2,3-diphosphoglyceric acid (2,3-DPG). Acute HP has been described as causing a variety of symptoms, such as cardiac dysfunction (cardiomyopathy, arrhythmias), respiratory failure, neurologic complications (delirium, seizures, encephalopathy, peripheral neuropathy), and haematologic complications (impaired oxygen release, haemolysis, leukocyte dysfunction).

A reduction in available ATP for respiratory muscle contraction has been suggested as a mechanism for acute respiratory failure. With a reduction in red blood cell 2,3-DPG, the affinity of the red cells for oxygen increases, the cells being less able to release oxygen to the tissues. The red cells become rigid and, therefore, more susceptible to lysis. Severe depletion of ATP levels in white blood cells impairs pseudopod and vacuole formation, thus limiting extravascular migration and phagocytic function. Cardiac dysfunction is thought to be occurred because HP leads to depleted ATPase levels and then depressed myocardial sarcomere contractility, or to acute myocardial damage itself. The pathophysiology of neurological dysfunction is not certain but it has been suggested that the physiological effects of HP, such as increased oxyhaemoglobin affinity and haemolytic anaemia, could lead to tissue hypoxia to altered tissue function (9).

Our first case as described above was particularly severe as mechanical ventilation was required to treat the respiratory failure that was associated with the severe hypokalaemia and moderate HP (Phosphate 2.1 mg/dL). Both second and the third cases presented with severe sepsis, hypokalaemia and severe HP (Case 2: P 0.9 mg/dL, Case 3: P 1.1 mg/dL). All the cases manifested obvious lethargy and hypotonia. It is very difficult to distinguish how much HP contributed to these symptoms because hypokalaemia and sepsis are also known to cause lethargy and hypotonia. It is possible that the extreme presentation of these children was due to a combination of the factors described. It is, therefore, important to remember that HP can be seen in addition to the other electrolyte imbalances among malnourished children in the acute phase, and the combination can lead to significant morbidity.

### Management

None of these cases of HP was treated using phosphate supplementation but improved with milk-feeding and vitamin D supplementation alone. Similarly, Marvin *et al.* described in their case series of adult patients who had developed HP within seven days of starting parenteral nutrition that 75% of cases had recovered within 10 days without phosphorus supplementation (10). However, Schwartz *et al*. described that patients with sepsis and low serum phosphate levels were at a greater risk of developing cardiac arrhythmias; they recommended that phosphate supplementation be given in the early stages of sepsis as a preventative measure (11). Currently, it is widely recommended that treatment commence when a patient's phosphate level is less than 1 mg/dL or symptoms of HP, such as rhabdomyolysis, tremour, paresthaesia, ataxia, seizures, delirium, or coma, are present. Initial doses are 0.08-0.16 mmol/kg (5 mg/kg) over 6 hours by intravenous infusion and are repeated depending on the severity of the phosphate deficit, and stopped once the phosphate levels are greater than 1.5 mg/dL, any symptoms have subsided, or severe side-effects are reported. Side-effects of intravenous phosphate repletion are hypocalcaemia, metastatic calcification, hyperkalaemia (which is associated with potassium-containing supplements), volume excess, metabolic acidosis, and hyperphosphataemia (4).

### Conclusions

Phosphorus is an essential substance, and HP is well-described in rickets, refeeding syndrome, DKA, and chronic alcohol-abuse. However, acute HP among severely-malnourished children has not received much attention, and related information, such as prevalence, severity, and treatment, is scarce. HP manifestations are diverse and the clinical picture is complicated by the presence of other electrolyte imbalance and micronutrient deficiencies. Malnutrition combined with sepsis is one of the major causes of mortality in children below five years of age, and both malnutrition and sepsis can cause HP. To reduce the mortality of children aged less than five years, we need to search actively for the underlying causes of their morbidity such as HP and should be treated. Finally, we suggest further study on HP among malnourished children.

## ACKNOWLEDGEMENTS

This study was funded by icddr,b and University of Tsukuba, Japan. icddr,b acknowledges with gratitude the commitment of the University of Tsukuba to its research efforts. It also gratefully acknowledges the following donors which provide unrestricted support: Australian Agency for International Development (AusAID), Government of the People's Republic of Bangladesh, Canadian International Development Agency (CIDA), Swedish International Development Cooperation Agency (Sida), and the Department for International Development, UK (DFID).
